# A rare occurrence of psoas abscess with uterine didelphys and renal agenesis: A case report

**DOI:** 10.1016/j.amsu.2021.102802

**Published:** 2021-09-04

**Authors:** Sujit Kumar Mandal, Shreeja Shikhrakar, Binit Upadhaya Regmi, Shiba Bam

**Affiliations:** a Department of Obstetrics and Gynecology, Nepalese Army Institute of Health Sciences-College of Medicine, Kathmandu, Nepal; b Department of Obstetrics and Gynecology, Kathmandu University School of Medical Sciences, Dhulikhel, Nepal

**Keywords:** Uterine didelphys, Renal agenesis, Psoas abscess, Mullerian duct anomaly, Case report

## Abstract

**Introduction:**

Mullerian duct anomaly such as uterine didelphys is a rare congenital anomaly of female genitourinary tract presenting with dysmenorrhea, hematocolpus, and subfertility in adolescent females. It is commonly associated with wolffian duct anomaly like renal agenesis. Here we present a rare association of psoas abscess with uterine didelphys and renal agenesis in a 21-year-old unmarried female.

**Case presentation:**

We report a case of 21-year-old female presenting with right hip pain, recurrent genital infections and dysmenorrhea. Her labs suggested infectious etiology whereas radiological investigation revealed right psoas abscess. In addition, she was found to have uterine didelphys with pyometra, right adnexal mass, and right renal agenesis.

**Clinical discussion:**

Uterine didelphys commonly present with dysmenorrhea and hematocolpos along with various non-specific symptoms. Patients can develop psoas abscess secondary to uterine didelphys, but uterine didelphys presenting with psoas abscess is fairly rare. Psoas abscess on itself is a difficult condition to diagnose, more so when associated with rare uterine anomalies.

**Conclusion:**

This case highlights the possibility of psoas abscess as a primary presentation of Mullerian duct anomaly. Further, a differential of uterine didelphys should be considered in every reproductive age female presenting with recurrent pelvic infection.

## Highlights

• When a reproductive-age women presents with recurrent pelvic infections, a differential of Mullerian duct anomalies should always be sought out despite the absence of its classical symptoms.
• Uterine didelphys can also present with rectal, abdominal, or hip pain due abscess formation in related organs and surrounding communicating structures.
• Our case is an example of atypical presentation of uterine didelphys where the patient presented with psoas abscess which is rare in this entity.

## 1 Introduction

Uterine didelphys with ipsilateral renal anomaly is an uncommon congenital Mullerian anomaly. It commonly presents with progressively worsening dysmenorrhea, hematocolpos, dyspareunia, and sub-fertility in adolescent females [1,2]. This is due to the distension caused by efflux from the ipsilateral uterus on obstructed hemivagina. Occasionally, patients present with marked rectal, abdominal, or hip pain due abscess formation in related organs and surrounding communicating structures [[3], [4], [5]]. We present here a case of a 21-year unmarried female with uterine didelphys primarily presenting with recurrent pelvic infection and secondary sterile psoas abscess.

This work has been reported in line with the SCARE 2020 criteria [6].

## 2 Case presentation

A 21-years-old unmarried female presented with a gradually progressive, continuous, and dull-aching right lower quadrant abdominal pain. She complained of right hip joint pain which was gradually progressive, continuous, pricking in character, and was aggravated on movement and hip extension. She also complained of fever associated with chills. There was no history of urinary complaints suggestive of UTI and there was no history of trauma. She had a history of dysmenorrhea since she attained her menarche at the age of 14. She also mentioned the history of intermittent whitish foul-smelling PV discharge preceding and following menstruation. The patient had managed conservatively with analgesic and antibiotics for several months elsewhere. She denied having family history of similar illness or other genetic disorders and had no history of long-term use of any drugs. She did not have any known history of allergy.

On abdominal examination, right iliac fossa tenderness was present. On speculum examination, the cervix was not visualized due to profuse purulent discharge. Vaginal examination revealed a ridge-like structure which was felt within the cervix. Fullness and tenderness were also noted in the right fornix.

Her complete blood count was raised to 20,700/mm^3^ with neutrophils 68.5% and lymphocytes 5.6% suggestive of infectious etiology. She was anemic with hemoglobin 9.5g/dl. Her RFT was within normal limit. Urine and high vaginal swab culture showed no growth. Sonographic imaging of the pelvis showed double cervix and uterus suggestive of uterine didelphys. The right uterus was bulky and contained minimal endometrial collection of 2.9cmX1.5cm. The right ovary was bulky and edematous. Complex right adnexal mass of size 4.5cmX4.5cm was visualized. The right kidney was absent. Sonographic imaging of the right hip revealed the right psoas abscess. MRI pelvis findings were consistent with the USG findings. The diagnosis of uterine didelphys with psoas abscess, pyometra, and renal agenesis was confirmed.

**Fig. 1:**
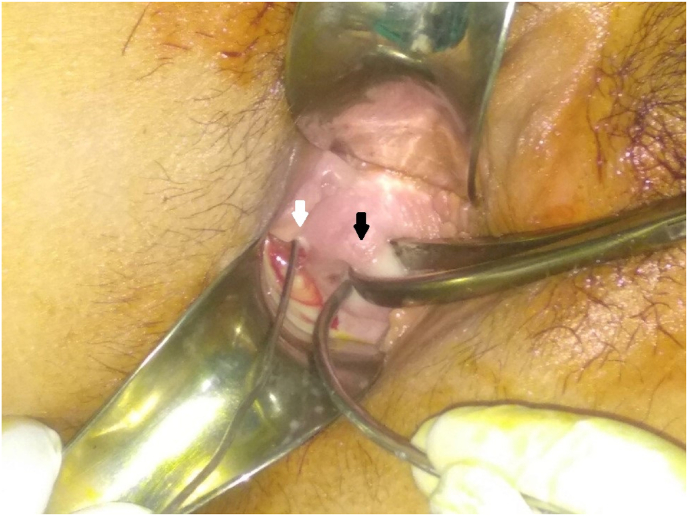
Figure showing normal vaginal canal and two cervices on per speculum examination under anesthesia. Left cervix (white arrow) was comparatively larger than the right (black arrow), with a uterine sound in-situ in the left cervical os and metallic catheter in-situ in the right cervical os.

On examination under anesthesia, pus was seen oozing out of pinpoint right os, whereas left os was normal. The left uterus could admit uterine sound up to 8 cm, whereas the right uterus could only admit a few small metallic wires of 4 cm (Fig. 1). Pus was drained using an infant feeding tube placed through the cervical os. Further, the psoas abscess was drained percutaneously using a pigtail catheter. The pus microscopy and culture were sent and the patient was managed with intravenous Ampicillin, Gentamicin, and Metronidazole post-operatively. On pus microscopic examination with gram stain and ZN stain, bacteria were not detected and on culture and sensitivity, growth was not seen. All of these findings pointed moreover towards the infection by atypical organisms. The patient improved symptomatically and her counts were reduced to 11,000/mm^3^. Furthermore, the patient was counseled about the possible infertility problems and gestational challenges like breech presentation, placenta previa, abruptio placenta, preterm labor, and a stillbirth. The management was done by consultant Gynecologists in assistance of gynecology residents at a tertiary care hospital in Nepal. On follow-up teleconsultation, the patient didn't have any symptoms and was not under any medication.

## 3 Discussion

Mullerian duct anomalies are disorders affecting the development of the female genitourinary system. Its incidence is believed to be 0.5–2.0% of reproductive-age women, and the didelphic uterus accounts for approximately 10% of all Mullerian duct anomalies. In this condition, failure of Müllerian ducts fusion leads to the formation of two separate uterine cavities, cervices, and vaginas [7]. As Müllerian ducts have close association and interrelated development with wolffian ducts, wolffian duct abnormalities such as congenital anomalies of kidneys and urinary tract are a common association. Occurrence of the characteristic triad of uterine didelphys, obstructed hemivagina and ipsilateral renal agenesis is termed as Herlyn Werner Wunderlich (HWW) syndrome or obstructed hemivagina and ipsilateral renal anomaly (OHVIRA) [8].

Uterine didelphys commonly present with dysmenorrhea and hematocolpos in adolescent females [3].however, presentation can be delayed when patients are asymptomatic or have mild symptoms. Recurrent pelvic infection in this age group should also lead us to suspect congenital anomalies of genital tract. In our case, the patient had dysmenorrhea since her menarche, and had recurrent vaginal discharge which was managed conservatively for several months before assessment for congenital anomalies. Late presentation is also possible with complaints of subfertility.

In addition to the non-specific symptoms, patients may rarely present with pelvic or hip joint pain due to the development of secondary psoas abscess. Psoas abscess in itself is a rare disease of varying etiology, and is difficult to diagnose and treat owing to its retroperitoneal location [9]. Due to their severe clinical course, they have a high mortality in the absence of timely intervention [10]. Moreover, psoas abscess secondary to uterine didelphys is rare, with hardly any case reported in the literature.

Diagnosis of uterine didelphys is done using invasive modalities such as hysteroscopy, hysterosalpingography, and laparoscopy, however, non-invasive modalities like an ultrasound can also help in visualizing the didelphic uterus. In addition, ultrasonography can also confirm the absence of one kidney and the presence of an obstructed genital tract [5,11].

Uterus didelphys has a good reproductive prognosis and needs intervention only when it is associated with obstructed hemivagina or HWW syndrome [12,13]. In those cases, one of the vaginas is obliterated and the septum needs to be surgically resected to allow the normal menstrual flow and restore the reproductive function. In our case, however, the didelphic uterus was not associated with obstructed hemivagina, and immediate surgical management as such was not warranted. In contrast, anticipation of future reproductive complications like abortion, pre-term delivery, breech presentation, placenta previa, abruptio placenta, and still birth, some literature suggests early hysteroscopic metroplasty in patients with uterine anomalies [14]. In this regard, our patient was comprehensively counselled about the long-term complications of this condition and their possible prophylactic intervention.

## 4 Conclusion

Uterine didelphys is commonly present with dysmenorrhea and hematocolpos in adolescent females. When a reproductive-age female presents with recurrent pelvic infection and secondary abscess of communicating structures, a differential of uterine anomalies should always be kept in mind. Patients should also be counseled about the possible infertility problems and gestational challenges along with the available prophylactic interventions. Preventing recurrence of genital infection is crucial in patients with uterine anomalies to prevent potentially life-threatening infections of adjacent pelvic structures.

### 4.1 Patient perspective

The patient was thankful for having her condition diagnosed and managed. She feels her symptoms have improved significantly and her insights regarding her condition has changed positively. Her concerns regarding the possibility of future obstetric complications ameliorated after comprehensive obstetric counselling.

## Provenance and peer review

Not commissioned, externally peer-reviewed.

## Authors’ contribution

Sujit Kumar Mandal and Shreeja Shikhrakar involved in manuscript writing, review and editing of the manuscript. Binit Upadhaya Regmi contributed in data collection and editing of the manuscript. Shiba Bam contributed in patient management and critically reviewing the manuscript for intellectual content.

## Sources of funding

The authors have not received any kind of financial aid in research and publication of this work.

## Ethical approval

No ethical approval was necessary for the given article.

## Consent

Written informed consent was obtained from the patient for publication of this case report and accompanying images. A copy of the written consent is available for review by the Editor-in-Chief of this journal on request.

## Registration of research studies

1. Name of the registry: N/A
2. Unique Identifying number or registration ID: N/A
3. Hyperlink to your specific registration (must be publicly accessible and will be checked): N/A

## Guarantor

Dr. Sujit Kumar Mandal

## Declaration of competing interest

The authors have no conflict of interest to declare.
